# A Comparison of Food Supply from 1984 to 2009 and Degree of Dietary Westernization in Taiwan with Asian Countries and World Continents

**DOI:** 10.1155/2015/628586

**Published:** 2015-07-29

**Authors:** Cheau-Jane Peng, Cheng-Yao Lin, How-Ran Guo

**Affiliations:** ^1^Department of Environmental and Occupational Health, College of Medicine, National Cheng Kung University, 138 Sheng-Li Road, Tainan 704, Taiwan; ^2^Office for Planning and Management, Chi Mei Medical Center, 901 Zhonghua Road, Yongkang District, Tainan 710, Taiwan; ^3^Department of Internal Medicine, Chi Mei Medical Center, 201 Taikang, Liouying District, Tainan 736, Taiwan; ^4^Department of Occupational and Environmental Medicine, National Cheng Kung University Hospital, 138 Sheng-Li Road, Tainan 704, Taiwan

## Abstract

*Objective*. To compare quality, quantity, and trends of food supply from 1984 to 2009 and degree of food westernization in Taiwan with Asian countries and world continents by using food balance data. *Methods*. We compiled data from food balance sheets of Taiwan and Food and Agriculture Organization, including five continents and three most populated countries each in Eastern, Southern, and Southeastern Asia over the period 1984–2009. Quantity of food supply per capita was referenced to Taiwan food guides. The population-weighted means of food supply from Europe, North America, South America, and Australia and New Zealand continents in terms of energy and nutrient distributions, animal/plant sources, and sugar/alcohol contribution were used as indicators of westernization. Trends of food supply per capita of six food groups were plotted, and linear regression was applied to evaluate food changes. *Findings*. Taiwan's food supply provided sufficient quantity in food energy, with the lowest cereals/roots supply and rice to wheat ratio, but the highest meat and oil supplies per capita among the 10 studied Asian countries. Taiwan food supply showed the most westernization among these countries. *Conclusion*. Food supply of Taiwan, although currently sufficient, indicated some security problems and high tendency of diet westernization.

## 1. Introduction

There is a need in every country for reliable sources of dietary data that can provide information for monitoring and identifying dietary and nutritional status of citizens. The food balance sheet (FBS) assembled by the Food and Agriculture Organization (FAO) of the United Nations [1] provides information on food supply at the population level, estimated on the basis of the annual food production, imports and exports, changes in stocks, and agricultural and industrial uses within a country, as well as losses during storage and transportation. FBS is used to monitor food availability as well as the trend of food supply to people over time, which may reflect favorable and undesirable changes of food habit [2, 3] in studies of prevalence of obesity [4], geographic comparison [5], and epidemiological characteristics [6]. FBS is one of the three most commonly used sources of dietary information, and when compared with the household budget surveys and individual dietary survey, FBS usually overestimates food consumption compared to the other two, owing to the representations in terms of different steps of the food chain [7]. However, these three sources may complement each other in dietary assessments at the population level [8].

According to the Global Burden of Disease Study 2010 [9], the highest impact from dietary risk factors was that from diets low in fruits, which contributed to 4.9 million (95% uncertainty intervals = 3.8–5.9) deaths and 4.2% (3.3–5.0) of disability-adjusted life years globally. There is also huge evidence supporting the relationship between poor diet components such as increase in saturated fat [10–12], total fat or energy from fat [13], and energy from animal foods [14–16], additive sugar [17–21], and alcohol [22–24] and adverse health outcomes such as weight gain, type 2 diabetes, heart disease, some cancers, and overall mortality.

“Western diet,” although without clear definition, is quite commonly used in describing the dietary pattern that corresponds to a diet low in dietary fiber but high in total fat, saturated fat, animal fat, and additive sugar [25], and the result would be nutritionally high fat, high protein, high animal fat, high animal protein, and low complex carbohydrate combined with high total energy and empty calories (defined as energy contributed by sugar and alcohol). Food habits that highlight the Western diet include high intakes of meats (especially red meat and processed meat), refined grains, high-fat dairy products, sweets, desserts, high-sugar drinks, and cheese and butter, as well as low intakes of whole grains, fruits, and vegetables [26]. Cardiovascular related disorders [27, 28] and colorectal cancer are well-proven negative impacts of western diet. A French study indicated that meat-eaters pattern was positively associated with a 58% increase in colorectal cancer [29], and a collective review reported that western diet increased cancer risk with odds ratios ranging from 1.26 to 3.48 [30]. Other studies demonstrated that western-like (or meat-based) diet patterns were positively associated with low-grade inflammation [31, 32], increased risks of obesity, type 2 diabetes [33], inflammatory autoimmune disease [34], inflammatory bowel disorders [35, 36], dementia [37], depression [38], impaired brain function [39], and so on. Although healthier diet recommendations have been advocated, western style diet is highly adopted in many countries, owing to its palatability and food globalization.

Taiwan has gone through rapid economic growth and is now one of the newly industrialized Asian economies [40]. The disease pattern also changed during these decades. The Nutrition and Health Survey in Taiwan in 2005–2008 (2005–2008 NAHSIT) demonstrated large growths in the prevalence of obesity, metabolic syndrome [41], and hyperuricemia [42] when compared with the previous survey in 1993–1996 (1993–1996 NAHSIT). The top 5 of the 10 leading causes of death in Taiwan in 2010 were malignant neoplasms, heart disease, cerebrovascular disease, pneumonia, and diabetes mellitus. The leading causes of death were not only turning from infectious to noninfectious chronic diseases, but were also more similar to the pattern of western developed countries.

Compared to the dietary patterns observed in the 1993–1996 NAHSIT, those observed in the 2005–2008 NAHSIT showed some changes that should be beneficial to health (increases in consumptions of fruit and vegetables, soy products, fish, whole grains, nuts, and seeds, accompanied by decreases in products made from animal fats and oils, red meat, and high sodium processed foods), while there were also some detrimental changes (increases in intakes of cakes, sweets, and sugary drinks) [43]. Those dietary changes interactively affect the food supply system around Taiwan. Since food availability data can be regarded as a surrogate of intake information at the population level, we conducted a closer evaluation of the quantity, quality, and trends indicated by food balance data in Taiwan and compared the information with neighborhood Asian countries as well as world continents, so that we can provide beforehand health inferences.

## 2. Method

FBS assembled by the Council of Agriculture of Taiwan was compiled with FAO food balance data of the whole world, six continents (Europe, Australia and New Zealand, North America, South America, Africa, and Asia), three most populated countries in Southeastern Asia (Vietnam, Indonesia, and Philippines), three most populated countries in Eastern Asia (Japan, [South] Korea, and [Mainland] China), and three most populated countries in Southern Asia (Bangladesh, India, and Pakistan). Taiwan had public accessible food balance data published from 1984 to 2010, while the FAO had the most updated international data published till 2009. As we calculated the 3-year moving averages, we hereby show data over the period of 1985 to 2008.

We examined Taiwan FBS data item by item to assign the codes of similar food from the FAO food balance data. For those food subgroup items without similar classification, we compared the total quantities rather than any single subgroup item. For example, Taiwan FBS listed green leaves, roots, bulbs and tubers, flowers and fruits, and mushroom as subgroups of the vegetables group, while FAO assigned the vegetable subgroup items as tomatoes, onions, beans, peas, aquatic plants, and other vegetables; therefore, we compared the whole vegetables group only, not the vegetable subgroup items.

The information on total energy (Kcal) and protein and fat (g) available per capita per day was compiled from FAO data and Taiwan FBS. We calculated the carbohydrate availability (g) by subtracting the sum of energy from fat and protein from the total energy and then dividing the number by 4. All single food items were further classified into one of the six food groups (cereals/roots, vegetables, fruits, dairy, soy/fish/meat/egg, and oil/nuts) as categorized by the Taiwan food guides. Except for the vegetables group, we calculated servings of food of their belonging food groups by one major nutrient quantity. In other words, items in the cereals/roots group that contain 15 g of carbohydrate were counted as one serving each, and it was 7 g of protein for soy/fish/meat/egg, 15 g of carbohydrate for fruits, 5 g of fat for oil/nuts, and 8 g of protein for dairy. For the vegetables group, an edible 100 g portion was counted as one serving [44].

The reference food needs per capita retrieved from the Taiwan food guides for adults were 12 servings of cereals/roots, 1 serving of dairy, 4 servings of soy/fish/meat/egg, 3 servings of vegetables, 2 servings of fruits, and 6 servings of oil/nuts. We used food availability in servings of the studied countries or continents as the indicator of food quantity.

We used the nutritional indices calculated as per capita food energy supplied from animal food (Kcal), total and animal protein (g), total and animal fat (g), contribution of animal-derived energy (%), contribution of animal protein (%), contribution of animal fat (%), contributions of protein and fat to total energy (%), and energy from sugar and alcohol (Kcal) as the indicators of food quality. This analysis was done on the 2007 data because we believe the stability before the 2008 economic crisis would give more reliable results. In addition, we determined the changes in food supply by using the slope (annual change) obtained from the linear regression model.

## 3. Results

### 3.1. Comparison of Quantity of Food Supply

**Figure 1:**
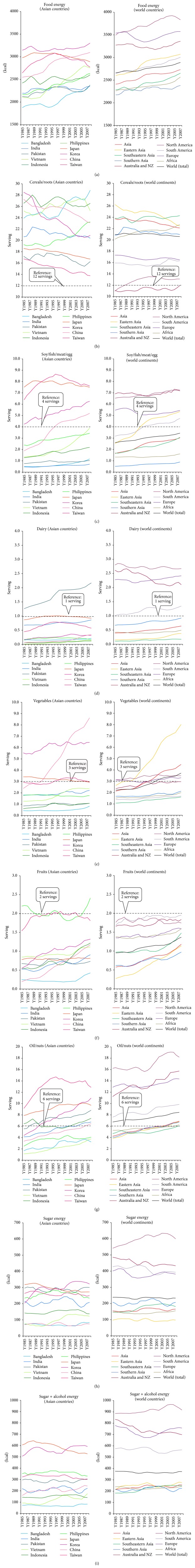
Food supply trends of total energy, the six food groups, sugar energy, and sugar + alcohol energy were plotted over the period 1984 to 2009. The six food groups plotted in the unit of servings are referenced to Taiwan food guides. Food items in the cereals/roots group that contain 15 g of carbohydrate were counted as one serving each, and it was 7 g of protein for soy/fish/meat/egg, 15 g of carbohydrate for fruits, 5 g of fat for oil/nuts, and 8 g of protein for dairy. For the vegetables group, an edible 100 g portion was counted as one serving. The numbers of food servings are also referenced to the Taiwan food guides. Data are presented as 3-year moving averages, and therefore the data were from 1985 to 2008.

Figure 1 demonstrated the trends of total energy, the six food groups, sugar energy, and sugar + alcohol supply of Taiwan compared with major Eastern, Southern, and Southeastern Asian countries and with world continents. For total energy supply, all studied Asian countries and the world continents in 2009 exceeded 2,100 Kcal/capita/day, which is the minimal nutritional target of the FAO [45]. Food energy supply of Taiwan had reached 3,000 Kcal/capita/day from 1993 to 2000 but had been dropping steadily since then. While this phenomenon was also seen in Japan, it was on the contrary to the continuously increasing trends in other Asian countries, the whole world, and all six continents.

For cereals/roots supply, all four Eastern Asian countries (Japan, Korea, China, and Taiwan) dropped steadily with the annual mean changes of −0.12, −0.24, −0.29, and −0.10 servings/capita/day, respectively. Taiwan and China showed similar rates of decrease, while Korea had faster and Japan had slower rates. Starting from 2003, Eastern Asian countries have had the mean cereals/roots supply falling below that of South Eastern Asian countries. Among all compared Asian countries, Taiwan had the lowest supply of cereals/roots in any single year throughout the study period.

Soy/fish/meat/egg supplied by most of the world continents kept an increasing trend, but Japan and Taiwan have been showing a decreasing trend since 1996. Albeit with a steady annual decrease of 0.08 servings/capita/day of meat, Taiwan had the highest meat supply per capita among Asian countries since 1990, and the number was even higher than that of any continent since 1986 and was twice that recommended by the Taiwan food guides.

Asian and western continents had obvious differences in dairy supply. Except for Japan, all of the Asian countries had mean dairy supply less than 1 serving/capita/day, much lower than that in Europe, North America, and Australia and New Zealand continents. In Taiwan, starting from 0.79 servings/capita/day in 1994, milk supply had decreased annually at a rate of 0.02 servings/capita/day. Till 2009, it decreased to as low as 0.58 servings/capita/day, which was 58% of the minimal recommended level.

Eastern Asia had a much faster increasing trend in vegetables supply than Southeastern Asia since 1990, with major contribution from the sharp increase of vegetable supply in China and steady high supply in Korea. All the Southeastern Asian countries had vegetables supply less than 2 servings/capita/day. Taiwan had approximately 3 servings/capita/day, which barely reached the reference vegetables needs for healthy adults.

Taiwan had the second highest fruit supply next to the Philippines, while it already fell below the recommended 2 servings/capita/day and kept an annual drop of 0.02 servings/capita/day. Korea and China, although low on fruit supply throughout the study period, showed significant annual increases of 0.03 and 0.05 servings/capita/day, respectively, which contributed significantly to the annual increase of 0.03 servings/capita/day in the whole world. It is worthy to note that all the world continents had fruit supply less than 2 servings/capita/day, the referenced fruit supply for healthy adults.

The oil/nuts supply had steadily increased in most of the Asian countries during the years before 1995 and thereafter showed a steady state in Taiwan, Japan, and Philippines. Taiwan had the highest edible oil supply among Asian countries, and the mean supply of 12.4 servings/capita/day observed in 2009 was twice the recommended oil/nuts serving of Taiwan food guides.

Sugar energy supply in most of the Asian countries (except for Bangladesh, Vietnam, Indonesia, and China) exceeded 200 Kcal/capita/day. When alcohol was added, the energy supply usually exceeded 300 Kcal. Japan (−5.1 Kcal) and Taiwan (−4.6 Kcal) had continuous annual dropping, while China (+6.1 Kcal) and Korea (+6.4 Kcal) had their annual increases in sugar + alcohol energy (kcal/capita/day) in the recent 10 years. The sugar + alcohol energy plotted in Figure 1 clearly showed that Asian countries had much lower values than those of any western continents.

### 3.2. Comparison of Quality of Food Supply

#### 3.2.1. Indices of Food Westernization

**Figure 2:**
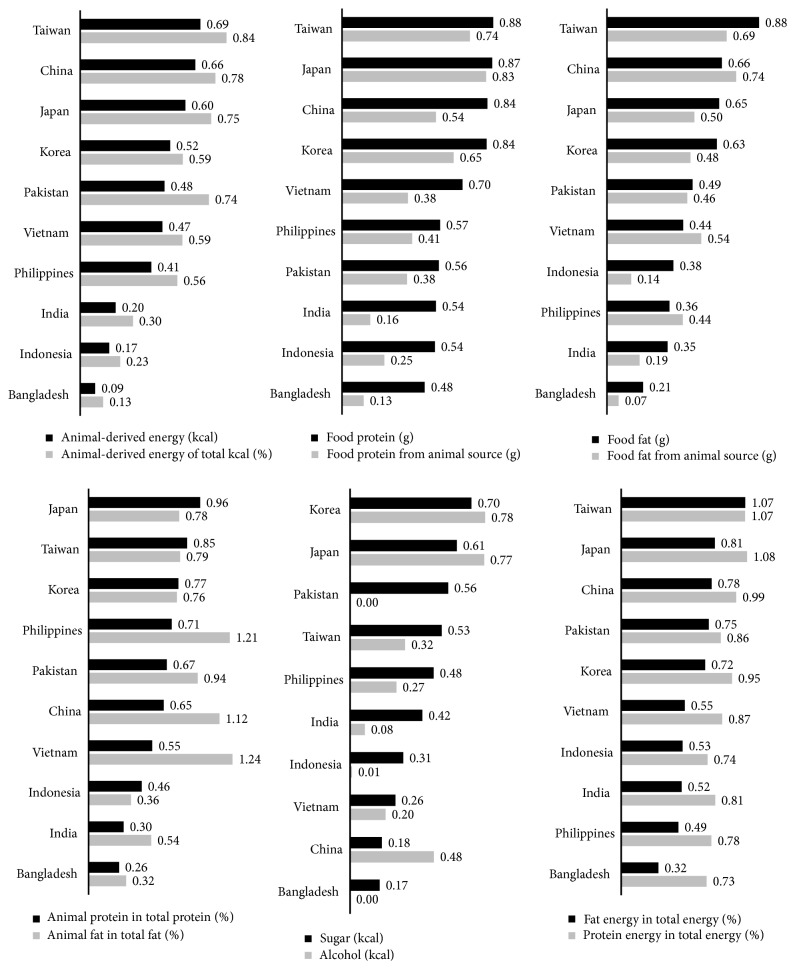
Comparison of indicators of food quality among 10 Asian countries. The numbers are ratios of indicators contrasted to Western-diet references, which were population weighted indicators of food quality from North America, Europe, and Australia & New Zealand food availability data. Panels are ranked by indicators of black bar.

Using FBS data in 2007, we found that the population-weighted data of Europe, North America, and Australia and New Zealand continents indicated a total energy of 3,300 Kcal/capita/day, with 25.5% of energy from animal food, 33.2% from fat, 11.8% from protein, 13.5% from sugar, and 4.3% from alcohol. The total food protein was 97 g/capita/day, with 54.5 g from animal food, and the total food fat was 122 g/capita/day, with 60.6 g from animal food. Sugar and alcohol contributed 446 and 142 Kcal/capita/day, respectively. We used these data as reference indices of food westernization.  Figure 2 plotted these indices of Taiwan and Asian countries, ranking from high to low.

Among the indices, Taiwan had the highest food energy from animal food, contribution of animal food to total energy, total food protein, total food fat, contribution of fat to total energy, and contribution of protein to total energy. In addition, next to Japan, Taiwan had the second highest food protein from animal sources, animal protein in total protein, and protein energy in total energy. In fact, Taiwan also had the second highest animal fat, next to China. Judging from the data above, we believe Taiwan had the most westernized food patterns among the Asian countries studied. Nonetheless, in Taiwan, the contribution of sugar + alcohol (empty calorie) to total energy was less than 53% of that in the western reference, indicating a less severe loading of those two components in Taiwan's dietary environment in comparison with western countries.

#### 3.2.2. Comparison of Food Source and Plant-to-Animal Ratios

Table 1 listed the serving quantities of the six food groups and some important subgroup food items, which were derived from 2007 FBS data. In general, Eastern Asian countries had higher total energy, food protein fat, and energy contributions from protein and fat than Southern and Southeastern Asian countries. In total food energy, Taiwan had the highest contributions from protein and fat and the lowest contribution from carbohydrate, even less than 50%. As for plant-to-animal ratios, Taiwan had the lowest plant-to-animal energy ratio and the second lowest (next to Japan) plant-to-animal protein ratio. Taiwan also had the highest plant oil supply and the second lowest (next to China) animal fat supply. Among the rice dominant Asian countries, food supply in Taiwan provided the lowest rice serving and rice-to-wheat ratio. On the other hand, Taiwan had the highest soy/fish/meat/egg supply, which was contributed by the highest (3.56) servings of domestic animals, the highest (2.11) servings of soy products, and the third highest (1.6) servings of seafood.

## 4. Discussion

FBS data represent the abundance or availability of food supply to people at the population level, without taking into consideration the issue of distribution among subgroups of the population. It is generally believed that FBS data overestimate food consumption compared with other dietary assessment methods. The difference may arise from household or business waste and spoilage, as well as food used for purposes other than human consumption. Thus, if FBS data showed a food supply that is equal to or lower than the recommended quantity, it should be regarded as “not adequate.” From our analysis, vegetables supply of Taiwan would be regarded as barely adequate, fruits as suboptimally adequate, and dairy as not adequate, while soy/fish/meat/egg and oil/nuts were adequate. Cereals/roots supply dropped steadily and had been not much higher than the minimal recommended level.

The data of food supply in Taiwan during the studied period showed that food energy had increased and then decreased, and similar trends were observed in soy/fish/meat/egg and dairy supplies; cereals/roots supply showed a constant decreasing trend; oil/nuts had a steep increase and then stayed constant; and vegetables and empty calorie (sugar + alcohol) supplies had slightly decreasing trends.

In comparison with other Asian countries, Taiwan had the highest food energy from animal food, protein supply, fat supply, contribution of fat to total energy, protein food servings (particularly from the soybean and the domestic animal subgroups), total oil servings, and plant oil servings but had the lowest contribution of carbohydrate to total energy, cereal servings, and rice-to-wheat ratio.

When the population-weighted food supply data of Europe, Australia and New Zealand, and North America Continents were selected as references to evaluate the degree of “westernization” of food pattern in Asian countries, Taiwan showed more westernization than the other Asian countries. On the other hand, Taiwan had some unique characteristics that would be beneficial to health, including higher supplies of soybean and seafood; relatively ample fruits and vegetables (although not ideally high); a higher plant oil to animal fat ratio; and a sugar + alcohol supply that was not too high. Because the food environment interacts with dietary habits of the population, those beneficial factors remaining in the Taiwan food supply system are worthy of further studies. Specifically, the impacts on health from the mixture of westernized factors and beneficial factors in Taiwan should be investigated.

## 5. Conclusion

From the evaluation of FBS over the period of 1984 to 2009, we found that food supply in Taiwan provided low dairy, suboptimal adequate fruits, barely adequate vegetables, constantly decreasing cereals/roots, and sufficient meats and oil/nuts. Compared to those in other Asian countries, Taiwan food supply provided higher animal food, meat servings, and animal protein and fat, but lower cereals/roots servings. It had the lowest plant-to-animal energy ratio, rice servings, and rice-to-wheat ratio, but the highest soy/fish/meat/egg supply. While food westernization in Taiwan is evident, there are also some characteristics that are beneficial to health such as higher supplies of soybean and seafood, relatively ample fruits and vegetables, a higher plant oil to animal fat ratio, and a sugar + alcohol supply that was not too high. These characteristics may alter the health impacts from pure western diet.

The understanding and continuous monitoring of food environment of Taiwan are helpful for food and nutrition research, policy, and education. Further investigation on food supply to health issues is also needed.

## Figures and Tables

**Table 1:** Characteristics of food supply of 10 Asian countries using 2007 food balance sheet data.

	Southern Asia			Southeastern Asia			Eastern Asia			
Bangladesh	India	Pakistan	Vietnam	Indonesia	Philippines	Japan	Korea	China	Taiwan

Energy									
Energy (Kcal)	2277	2365	2290	2853	2538	2606	2944	3224	3071
Macronutrients									
Protein (g)	50.6	57.5	58.9	73.5	56.5	59.7	92.6	91.3	89.6
Fat (g)	28.4	48.0	68.2	60.6	53.2	49.3	89.3	87.5	91.1
Carbohydrate (g)	456	427	362	504	460	482	445	521	475
Energy distribution									
Protein (%)	8.9%	9.7%	10.3%	10.3%	8.9%	9.2%	12.6%	11.3%	11.7%
Fat (%)	11.2%	18.3%	26.8%	19.1%	18.9%	17.0%	27.3%	24.4%	26.7%
Carbohydrate (%)	80.1%	72.3%	63.2%	70.6%	72.5%	73.9%	60.5%	64.6%	61.9%
Plant-animal ratio									
Plant-derived energy (Kcal)	2194	2168	1823	2396	2379	2213	2361	2722	2433
Animal-derived energy (Kcal)	83	197	467	457	159	393	583	502	638
Plant-to-animal energy ratio	26.4 : 1	11.0 : 1	3.9 : 1	5.2 : 1	15.0 : 1	5.6 : 1	4.0 : 1	5.4 : 1	3.8 : 1
Plant-derived protein (g)	42.9	47.2	35.6	49.7	41.3	34.4	40.2	50.9	55.5
Animal-derived protein (g)	7.7	10.3	23.3	23.8	15.2	25.3	52.4	40.4	34.1
Plant-to-animal protein ratio	5.6 : 1	4.6 : 1	1.5 : 1	2.1 : 1	2.7 : 1	1.4 : 1	0.8 : 1	1.3 : 1	1.6 : 1
Plant oil (g)	23.8	34.9	35.8	22.6	43.3	18.9	53.7	53.9	39.1
Animal fat (g)	4.6	13.1	32.4	38.0	9.9	30.4	35.6	33.6	52.0
Plant-to-animal oil/fat ratio	5.2 : 1	2.7 : 1	1.1 : 1	0.6 : 1	4.4 : 1	0.6 : 1	1.5 : 1	1.6 : 1	0.8 : 1
Staples									
Rice (serving)	24.0	10.6	2.2	24.1	18.5	19.3	9.2	12.5	11.9
Wheat (serving)	1.8	7.2	12.0	1.6	2.3	2.1	5.1	5.5	8.2
Rice-to-wheat ratio	13.6 : 1	1.5 : 1	0.2 : 1	15.0 : 1	8.2 : 1	9.3 : 1	1.8 : 1	2.3 : 1	1.5 : 1
Protein food									
Domestic animal (serving)	0.23	0.20	0.79	2.07	0.66	1.60	2.33	2.67	2.67
Seafood (serving)	0.60	0.23	0.07	1.01	1.14	1.47	3.16	2.06	0.94
Eggs (serving)	0.06	0.09	0.10	0.11	0.23	0.26	0.89	0.47	0.79
Soybeans (serving)	0.13	0.11	0.00	0.30	0.17	0.01	1.23	0.79	0.50
Domestic animal-to-seafood ratio	0.4 : 1	0.9 : 1	11.0 : 1	2.0 : 1	0.6 : 1	1.1 : 1	0.7 : 1	1.3 : 1	2.8 : 1
Food guides (serving)^*^									
Cereal/roots	27.4	21.0	15.7	27.6	25.8	23.3	16.8	20.2	23.2
Dairy	0.19	0.84	2.08	0.15	0.13	0.21	0.95	0.34	0.34
Vegetables	0.71	2.15	1.06	2.38	1.05	1.68	3.01	6.24	7.91
Fruits	0.38	0.86	0.71	1.07	1.20	2.29	0.82	1.35	1.13
Soy/fish/meat/egg	1.01	0.63	0.96	3.50	2.20	3.34	7.63	6.14	4.99
Oil/nuts	3.6	6.7	8.2	3.4	7.2	3.9	10.0	10.3	6.7

^*^Food serving numbers were referenced to the Taiwan food guides. Food items in the cereals/roots group that contain 15 g carbohydrate were counted as one serving each, and it was 7 g of protein for soy/fish/meat/egg, 15 g carbohydrate for fruits, 5 g fat for oil/nuts, and 8 g of protein for dairy. For the vegetables group, an edible 100 g portion was counted as one serving.
